# The relationship between health literacy and healthy lifestyle behaviors of women with preeclampsia

**DOI:** 10.1590/1806-9282.20241801

**Published:** 2025-06-16

**Authors:** Merve Kolcu, Nur Bahar Kuru Aktürk, Gizem Öztürk, Mine Akkuş

**Affiliations:** 1 University of Health Sciences Türkiye, Hamidiye Faculty of Nursing, Department of Public Health Nursing – İstanbul, Türkiye.; 2 Istanbul Arel University, Faculty of Health Sciences – İstanbul, Türkiye; 3 Fenerbahce University, Faculty of Health Sciences, Department of Nursing – İstanbul, Türkiye.; 4 Zeynep Kamil Women and Child Diseases Training and Research Hospital – İstanbul, Türkiye.

**Keywords:** Health behavior, Health literacy, Preeclampsia, Pregnancy, Woman

## Abstract

**OBJECTIVE:**

The aim of this study was to explore the relationship between the levels of health literacy and healthy lifestyle behaviors in women with preeclampsia. This research was planned as a descriptive relational study.

**METHODS:**

The data were collected from 251 pregnant women with preeclampsia who were admitted to a training and research hospital between February and July 2024. The Patient Information Form, Health Literacy Scale, and Healthy Lifestyle Behaviours in Pregnancy Scale were used for data collection.

**RESULTS:**

The mean Health Literacy Scale total score of the women was 76.25±14.48, and the mean Healthy Lifestyle Behaviours in Pregnancy Scale total score was 110.17±17.57. A moderate positive correlation was found between the mean total scores of the scales (r=0.365, p<0.05).

**CONCLUSION:**

As health literacy levels increased among women diagnosed with preeclampsia, the level of healthy lifestyle behaviors also increased. It would be beneficial to integrate the topic of health literacy into prenatal care and pregnancy preparation classes.

## INTRODUCTION

Preeclampsia (PE) represents a significant public health concern with a detrimental impact on maternal morbidity and mortality rates in developing countries. It is responsible for 14% of preventable maternal deaths globally and 15.5% in Türkiye^1,2^.

Health literacy (HL) encompasses "the ability to obtain, process, and understand the basic information and services necessary for individuals to make the right decisions about health"^3^. The literature reveals that women with inadequate HL tend to have limited knowledge about PE and are more likely to have an inadequate understanding of prenatal screening tests^4^. It is, therefore, crucial to increase women's HL level to facilitate positive developments in the health of women, infants, and communities^5^.

Healthy lifestyle behaviors (HLBs) influence a woman's approach to pregnancy and become a factor that determines the course of the pregnancy^6^. The World Health Organization has indicated that approximately three-quarters of maternal deaths in developed countries and almost half in the least developed or developing countries are attributable to negative health behaviors^7^.

The literature on health perception and HL in pregnant women is relatively limited^8^. To the best of our knowledge, this is the first study addressing the relationship between the levels of HL and HLBs in women with PE. Therefore, this study aimed to determine this relationship. It is believed that this study will provide a valuable guide for future studies in this field.

## METHODS

### Study participants

This study employed a descriptive correlational design to examine the relationships between variables and reported following the STROBE guidelines^9^. Data were collected from women who were admitted to the gynecology and obstetrics clinic of a training and research hospital between February and July 2024. An average of 120 women meeting the study's inclusion criteria visit the hospital monthly. Using this figure as the population, the minimum sample size was calculated to be 251, with a 95% confidence interval. Thus, a total of 251 women constituted the sample of the study. The inclusion criteria were determined as follows: (a) voluntary participation, (b) diagnosed with PE, (c) aged between 18 and 49 years, and (d) currently pregnant.

The three questions were as follows:

- What is the level of HL of women with PE?
- What is the level of HLBs of women with PE?
- Is there a relationship between the levels of HL and HLBs in women with PE?

### Data collection

A researcher conducted in-person interviews with participants who provided informed consent. The data collection was completed when the sample size was reached. The data were collected using the Patient Information Form, Health Literacy Scale (HLS), and Healthy Lifestyle Behaviours in Pregnancy Scale (HLBPS):

Patient Information Form: This form was created by the researchers and consisted of 15 questions. The form included questions related to sociodemographic characteristics, pregnancy, and PE^10^.

The HLS: This was originally developed by Sørensen et al., and its revised version was adapted for the Turkish population by Aras and Bayık Temel^11,12^. The scale consists of a total of 25 items and four sub-dimensions (access to information, understanding information, evaluation, and application) and is a 5-point Likert type. The scale's total scores range from 25 to 125. A low score indicates insufficient HL, while a high score indicates sufficient HL. The scale's internal consistency alpha coefficient was α=0.92 in the original study and α=0.90 in the current sample.

The HLBPS: This scale was developed by Yılmaz and Karahan^13^. The scale is of a 5-point Likert type and can be applied to all pregnant women. It consists of 29 items and six subscales (pregnancy responsibility, nutrition, hygiene, physical activity, travel, and acceptance of pregnancy). The scale items are scored between 1 and 5, with "always (5)" and "never (1)" as the options. There are no reverse-coded items in the scale, and higher scores indicate that the level of HLBs is positive/high. The scale's internal consistency was α=0.83 in the original study and α=0.84 in the current sample.

### Statistical analysis

The data were analyzed via IBM SPSS 25.0. Descriptive statistics and Pearson's correlation analysis were used. The confidence interval was considered 95%. The internal consistency coefficient (Cronbach's alpha) was calculated.

Ethical consideration: This study adhered to the ethical principles outlined in the Declaration of Helsinki. Ethical approval was received from the university's ethics committee (date-number: 11.10.2023-136) and the hospital (date-number: 17.11.2023-229599434).

## RESULTS

Among women diagnosed with PE, 61% were 35 years or older. Regarding socioeconomic factors, 27.9% were high school graduates, 66.5% had a moderate economic status, and 67.7% were not working.

The clinical characteristics included a high prevalence of overweight and/or obesity (92%) and elevated blood pressure, with a mean systolic blood pressure of 122.58±10.62 mmHg and a mean diastolic blood pressure of 78.57±9.06 mmHg. Most women (86.5%) expressed a desire for their current pregnancy. The majority had no history of miscarriages (68.9%) or stillbirths (89.6%), and 90% had no family history of PE. In addition, most women (83.3%) had no chronic disease, 70.5% were nonsmokers, and 84.1% did not consume alcohol (Table 1).

**Table 1:** Participants’ demographic characteristics (n=251).

Characteristics		n	%
Age (years)			
	18–24	32	12.7
	25–34	66	26.3
	35 and above	153	61.0
Education level			
	Primary school	56	22.3
	Secondary school	57	22.7
	High school	70	27.9
	Undergraduate degree	68	27.1
Economic status			
	Good	22	8.8
	Moderate	167	66.5
	Poor	62	24.7
Employment			
	Yes	81	32.3
	No	170	67.7
BMI (kg/m²)			
	Normal weight	20	8.0
	Overweight	83	33.1
	Obesity Class I	86	34.3
	Obesity Class II	50	19.9
	Obesity Class III	12	4.8
Desiring the current pregnancy			
	Yes	217	86.5
	No	34	13.5
History of miscarriages			
	Yes	78	31.1
	No	173	68.9
History of stillbirths			
	Yes	26	10.4
	No	225	89.6
Preeclampsia in family history			
	Yes	25	10.0
	No	226	90.0
Chronic disease			
	Yes	42	16.7
	No	209	83.3
Smoking			
	Yes	33	13.1
	No	177	70.5
	Before pregnancy	41	16.3
Alcohol consumption			
	Yes	4	1.6
	No	211	84.1
	Before pregnancy	36	14.3
Blood pressure		Min–Max	M (SD)
Systolic blood pressure (mmHg)		90–170	122.58 (10.62)
Diastolic blood pressure (mmHg)		60–120	78.57 (9.06)

n: sample size; %: percentage; BMI: body mass index; normal weight (18.5–24.9 kg/m^2^); overweight (25–29.9 kg/m^2^); Obesity Class I (30–34.9 kg/m^2^); Obesity Class II (35–39.9 kg/m^2^); Obesity Class III (40 kg/m^2^ and above); Min: minimum; Max: maximum; M: mean; SD: standard deviation.

Those with an undergraduate degree had a statistically significantly higher HLS total score compared to those with only a primary school education (p<0.05). Similarly, those with a family history of PE had a statistically significantly higher HLS total score compared to those without (p<0.05). It was also concluded that those with an undergraduate degree and those with a normal body mass index (BMI) had a statistically significantly higher HLBPS total score compared to those with only a primary school education and those classified as Obesity Class III, respectively (p<0.05) (Table 2).

**Table 2:** Comparison of sociodemographic characteristics with Health Literacy Scale and Healthy Lifestyle Behaviours in Pregnancy Scale total scores.

Characteristics		HLS total score		HLBPS total score	
Age (years)		Mean±SD	Statistical significance	Mean±SD	Statistical significance
18–24		75.21±15.25	F: 0.541	111.90±16.91	F: 0.183
25–34		75.79±14.57	p: 0.583	110.01±17.69	p: 0.833
35 and above		77.81±13.99		109.72±17.81	
Education level					
	Primary school	69.07±15.67	**F: 9.380**	101.83±17.08	**F: 6.414**
	Secondary school	74.32±14.55	**p: 0.000**	110.22±16.59	**p: 0.000**
	High school	78.62±12.57		112.58±16.32	
	Undergraduate degree	81.42±12.66		114.51±18.01	
Economic status					
	Good	73.54±13.82	F: 1.107	106.18±24.36	F: 1.508
	Moderate	77.19±14.43	p: 0.332	111.49±16.18	p: 0.223
	Poor	74.67±14.80		108.03±18.23	
Employment					
	Yes	76.29±15.46	t: 0.031	109.83±19.71	t: −0.209
	No	76.23±14.04	p: 0.975	110.33±16.51	p: 0.835
BMI (kg/m²)					
	Normal weight	79.90±11.55	F: 0.872	113.70±16.21	**F: 2.983**
	Overweight	76.04±13.76	p: 0.481	113.40±19.28	**p: 0.020**
	Obesity Class I	74.61±14.89		112.67±15.59	
	Obesity Class II	78.30±16.42		110.50±14.39	
	Obesity Class III	74.83±11.91		105.02±18.21	
Desiring the current pregnancy					
	Yes	76.53±14.56	t: 0.759	110.76±18.01	t: 1.335
	No	74.50±14.06	p: 0.448	106.44±14.07	p: 0.183
History of miscarriages					
	Yes	75.47±13.06	t: −0.573	107.74±17.94	t: −1.476
	No	76.60±15.10	p: 0.567	111.27±17.34	p: 0.141
History of stillbirths					
	Yes	75.11±14.20	t: −0.423	111.65±13.76	t: 0.452
	No	76.38±14.54	p: 0.673	110.01±17.97	p: 0.651
Preeclampsia in family history					
	Yes	82.36±12.72	**t: 2.239**	113.01±10.61	t: 0.847
	No	75.57±14.53	**p: 0.026**	109.86±18.16	p: 0.398
Chronic disease					
	Yes	78.64±13.09	t: 1.172	109.71±18.69	t: −0.186
	No	75.77±14.73	p: 0.242	110.26±17.38	p: 0.853
Smoking					
	Yes	76.39±12.70	F: 0.031	110.93±17.32	F: 0.535
	No	76.11±13.98	p: 0.969	110.63±17.46	p: 0.587
	Before pregnancy	76.73±17.92		107.58±18.43	
Alcohol consumption					
	Yes	73.75±10.68	F: 2.913	100.01±16.85	F: 0.783
	No	75.39±14.45	p: 0.056	110.54±17.27	p: 0.458
	Before pregnancy	81.58±14.48		109.11±19.42	

F: one-way ANOVA; t: independent t-test; HLS: Health Literacy Scale; HLBPS: Healthy Lifestyle Behaviours in Pregnancy Scale; SD: standard deviation; BMI: body mass index. Statistically significant values are denoted in bold.

The mean total score of the HLS was 76.25±14.48. The subscale scores were as follows: access information (14.83±3.58); understand information (21.52±4.79); appraise information (24.32±5.18); and apply information (15.57±3.39). Furthermore, the participants demonstrated a mean total score of 110.17±17.57 on the HLBPS. The examination of the subscales of HLBPS yielded the following mean scores: pregnancy responsibility, 17.39±3.40; nutrition, 32.13±6.47; hygiene, 17.72±3.23; physical activity, 10.32±3.11; travel, 19.58±4.80; and accepting pregnancy, 16.65±3.30 (Table 3).

**Table 3:** Relationship between Health Literacy Scale and Healthy Lifestyle Behaviours in Pregnancy Scale.

Scale	Min–Max	M (SD)	HLS total score	HLBPS total score
HLS total score	25–100	76.25 (14.48)		**0.365** *
Access information	5–20	14.83 (3.58)		**0.320** *
Understand information	7–28	21.52 (4.79)		**0.298** *
Appraise information	8–32	24.32 (5.18)		**0.335** *
Apply information	5–20	15.57 (3.39)		**0.286** *
HLBPS total score	48–140	110.17 (17.57)		
Pregnancy responsibility	4–20	17.39 (3.40)	**0.270** *	
Nutrition	14–45	32.13 (6.47)	**0.266** *	
Hygiene	4–20	17.72 (3.23)	**0.308** *	
Physical activity	3–15	10.32 (3.11)	**0.190** *	
Travel	5–25	19.58 (4.80)	**0.334** *	
Accepting pregnancy	4–20	16.65 (3.30)	**0.256** *	

Max: maximum; Min: minimum; M: mean; SD: standard deviation; HLS: Health Literacy Scale; HLBPS: Healthy Lifestyle Behaviours in Pregnancy Scale;

* Pearson's correlation p<0.05.

Statistically significant values are denoted in bold.

Pearson's correlation analysis revealed a moderate positive correlation between the mean total HLS score and the mean total HLBPS score (r=0.365, p<0.05). The mean total HLS score showed a weak positive correlation with all the subscales of HLBPS (p<0.05): pregnancy responsibility (r=0.270), nutrition (r=0.266), hygiene (r=0.308), physical activity (r=0.190), travel (r=0.334), and accepting pregnancy (r=0.256). Additionally, the mean total HLBPS score demonstrated weak positive correlations with all HLS subscales (p<0.05): access information (r=0.320), understand information (r=0.298), appraise information (r=0.335), and apply information (r=0.286) (Table 3).

## DISCUSSION

The HL level of pregnant women can directly affect the health of both the woman and her baby during pregnancy. Previous research has established a positive correlation between the HL level of pregnant women and their HLBs^5,14,15^. A literature review revealed a research gap in the relationship between the levels of HL and HLBs in women with PE. Examination of this issue is important for identifying and monitoring at-risk groups. This study aimed to determine the relationship between the levels of HL and HLBs in women with PE.

Advanced maternal age is a significant risk factor for PE^16^. The incidence of PE increases with age, and the presence of chronic diseases further exacerbates this risk. In the present study, 61% of the women diagnosed with PE were aged 35 years or older. This finding aligns with previous research: Jancsura et al.^17^ reported that a maternal age of 35 years and older is associated with an increased risk of PE. Similarly, Ives et al.^18^ identified age of 40 years and older as a risk factor for this condition. Melchiorre et al. emphasized the increased risk of various pregnancy complications, including diabetes mellitus, placental detachment, and, notably, PE, in older pregnancies^19^.

A family history of hypertension is one of the obstetric risk factors. Previous studies have reported that the presence of hypertension or chronic disease in the family may constitute a risk factor for the development of PE during pregnancy. The presence of PE in the medical history of first-degree relatives increases the risk of PE two- to fourfold^18^. While 90% of the participants in this study had no family history of PE, Nayak reported that more than half of the pregnant women in their study had a family history of hypertension^20^. This discrepancy may be attributed to the greater mean age of the women in this study.

HL in women is crucial for protecting and improving individual and community health^5,15^. The mean HLS scores of women with PE were assessed as moderate, in this study. While HL is vital for all individuals, it is particularly critical for specific populations, including pregnant women and those with PE, as described in this study^15^. Although some studies have explored HL level in pregnant women, including those with specific conditions such as diabetes, no research has specifically focused on the HL level of women with PE^21^. A study investigating the relationship between preventive behaviors and HL level in pregnant women reported that almost half of the participants had marginal or adequate HL^14^. Furthermore, research on pregnant women with diabetes revealed that 22% had low HL^21^. Since HL levels vary widely, they may be influenced by factors like ethnicity and cultural characteristics. Understanding HL levels in women with PE is essential for tailoring health services effectively. Addressing HL needs can help healthcare professionals empower women to participate in their care, improving disease management and reducing postnatal complications.

It is stated that higher educational attainment among women has a positive impact on the health of both mothers and children. Educated women, by being more conscious and sensitive about health issues, can contribute to the reduction of maternal and infant mortality rates. In this study, it was also concluded that as the level of education increased, the HLS and HLBPS total scores increased. Similar studies support this finding as well^20-22^. Furthermore, in this study, it was observed that those with a normal weight had higher HLBPS total scores compared to those classified as Obesity Class III. As with other diseases, an increase in BMI during pregnancy creates a predisposition for the incidence of PE^15^. For these reasons, BMI assessment and maintaining healthy eating habits are extremely important for effective PE management in pregnant women.

In this study, the HLBs of women with PE were evaluated as high. Kırca et al.^22^ found that the level of HLBs and the quality of life of pregnant women were moderate. They also observed a correlation between these variables^22^. Other studies reported moderate-to-high quality of life in pregnant women^15,23^. The differences in levels observed may be due to sociocultural, individual, and pregnancy factors, which can significantly impact both HLBs and quality of life. Health professionals should provide comprehensive education and follow-up care to women with PE to support and improve both areas.

A moderate positive correlation was found between the mean total HLS score and the mean total HLBPS score. This result indicates that a higher HL level is associated with increased HLBs in women with PE. While no directly comparable study was found in the literature review, this finding aligns with research by Mogharab et al.^24^, who demonstrated that the quality of life improved as HL levels increased in pregnant women. Given the importance of HLBs in managing PE, this study highlights the need to prioritize HL enhancement in prenatal care. Therefore, assessing and improving HL level should be a key component of health services for pregnant women with PE.

## CONCLUSION

This study found that higher HL levels were linked to increased HLBs in women with PE, emphasizing the importance of enhancing HL level to manage PE and improve pregnancy outcomes. Given the limited research, further studies are needed to explore this relationship across diverse populations. To address inadequate HL level, HL education should be integrated into prenatal care, including pregnancy preparation classes. Promoting HL among women with PE is crucial for improving healthcare quality and empowering women to actively engage in their care.

## Data Availability

The data of the study are available upon reasonable request.
